# Physical Fitness in Young Padel Players: A Cross-Sectional Study

**DOI:** 10.3390/ijerph18052658

**Published:** 2021-03-06

**Authors:** Javier Courel-Ibáñez, Javier Llorca-Miralles

**Affiliations:** 1 Department of Physical Activity and Sport, Faculty of Sport Sciences, University of Murcia, 30100 Murcia, Spain; 2 FIDIAS Sport & Health Center, Puerto de Santa María, 11500 Cádiz, Spain; llorcamiralles@gmail.com

**Keywords:** racket sports, exercise, paddle tennis, healthy growth, leisure-time physical activity

## Abstract

This study aimed to examine the fitness characteristics and to identify the influence of gender and practice experience between young amateur padel players. A total of thirty-four padel players (*n* = 19 boys and 15 girls) aged 13 to 17 years old (age 14.6 ± 1.5 years; body mass 63.4 ± 14.5 kg; height 166.6 ± 9.8 cm; 6.2 ± 2.5 padel experience) volunteered to participate. Body composition was assessed by bioimpedance. Change of direction and agility were evaluated by two padel-adapted tests. Upper-limb strength measurement included overhead and side medicine ball throws with dominant and non-dominant hands. One-way ANCOVA was used to determine whether there were significant differences between gender and experience on fitness variables adjusting for age as a covariate. Male and female young padel players presented an apparently healthy body composition and exhibited similar performance in all fitness tests except for jumping ability. Practice experience seemed to influence upper-limb throwing strength, however, sub-analyses revealed no conclusive results. These results contribute to the existing knowledge in padel by providing new data about the fitness status of amateur young players aged 13 to 17 years old and open a window for future interventions using padel as a health promotion tool among youths.

## 1. Introduction

In recent times, padel has become a worldwide popular racket sport, counting with Federative support in over 40 countries [1]. The rising fame of padel can be attributable to its intermittent and explosive nature, with high-frequent, high-intensity actions (~4–6 strokes per rally, for a total of ~300 hits per game), taking place around a small, enclosed court of 10 × 20 m in which the ball can rebound on the side and back walls [2,3]. These characteristics result in an increased game rhythm and the number of actions per point, but without increasing physical intensity compared to similar racket sports [4,5,6]. In addition, regular padel practice may have important advantages compared to other racket sports that make it a powerful tool for health promotion. For instance, it does not need high technical skills to start practicing, the high frequency of actions and long duration of rallies increases enjoyment, it can be played outdoor and it requires accessible equipment [7,8,9]. Hence, padel seems to play an important role in promoting physical activity and healthy habits while limiting sedentary behaviour. This is particularly relevant in youths considering that insufficient physical activity has raised the levels of unhealthy body composition, overweight, and obesity at epidemic proportions with an alarming increase of ten-fold over the last forty years [10].

The increasing interest in padel has led to many studies concerning match activity [6,9,11,12], players’ movements and distance covered on the court [4,13,14], game technical-tactical dynamics [5,15,16,17,18], fitness status [19,20,21], and injuries [22,23,24]. However, most of the available literature involved professional players or adult samples and little is known about young padel competition particularities. Recent research has described the game response and match activity in high-level, young players aged 14 to 17 years, finding longer rallies, longer resting interval time, and more number of strokes per rallies compared with other racket sports, resulting in a lower effort index [6]. Arguably, these lower physical demands could be insufficient to promote meaningful changes in fitness status and health. Conversely, recent studies in adult samples suggest that padel practice is associated with better fitness conditioning, body composition, strength, and balance [19]. However, the extent to which regular padel practicing may benefit fitness in young players remains unknown.

Only one previous study has examined fitness level in young padel players, testing a small sample of boys and girls aged 11 to 16 years [25]. In this first approach, authors identified gender differences with boys performing faster 10-m and 20-m sprints and having greater throwing strength (overhead medicine ball throw) than girls. Whereas 10-m and 20-m sprint tests are commonly used in sport, the small size of the padel court and the fact that players mostly made diagonal or lateral movements [4] and covered less than 8 m running [26] suggest a relevance of short running bouts and agility rather than straight sprints in padel. In this sense, other authors proposed using a more practical and specific agility test for padel [27], which merits further examination. Similarly, in addition to the overhead medicine ball throw, the assessment of swinging actions by rotational medicine ball throws would be advisable to describe strength performance in racket sports such as padel [28,29].

A better understanding of the physical profile of competitive players is also essential for improving the padel training process and talent scoping [30,31]. Information about how players respond to particular tests assists coaches in setting benchmarks and developing effective training drills accordingly [32]. However, information about fitness determinants in young padel players is very limited. Based on available information, some gender differences could be expected, with boys showing a higher strength and sprint performance. Therefore, this study aimed to examine the fitness characteristics and to identify the influence of gender and practice experience between young padel players.

## 2. Materials and Methods

### 2.1. Experimental Design

This is a cross-sectional study conducted in a Spanish padel club, with tests performed in an outside padel court (grass surface and glass walls). Before the evaluations, players underwent a familiarization session and a body composition assessment. Testing sessions included a neuromuscular test battery consisted of jumps, medicine ball throws, and change of direction/agility measurements. Participants were asked to refrain from vigorous activity for 48 h before the experiment. Experimental procedures were performed in the morning (10:00 a.m). Environmental conditions were measured using a portable weather station (WMR 108, Mextech, Mumbai, India) and the Windy app for Android, with all the measurements made under similar conditions (~27 °C, ~65% air humidity, wind < 5 km∙h^−1^). Testing sessions started with a 15-min standardized dynamic, low- to moderate-intensity (self-perceived), warm-protocol consisting of 5 min of jogging, 5 min of joint mobility, 2 × 15 m progressive accelerations with 1 min of rest between, 5 progressive jumps, and 1 practice trial for each test. These procedures have been reviewed and approved by the University Bioethics Commission which complied with the recommendations of the Declaration of Helsinki (ID: 2421/2019, data of approval: 22 May 2019) [33].

### 2.2. Participants

Power analysis was conducted to determine the required sample size to identify significant differences with a power ~0.80 and alpha level ~0.05. A total of thirty-four padel players (*n* = 19 boys and 15 girls) aged 13 to 17 years old (age 14.6 ± 1.5 years; body mass 63.4 ± 14.5 kg; height 166.6 ± 9.8 cm; 6.2 ± 2.5 years of practice) volunteered to participate in this study. Active players with a minimum of 2 years of experience competing at the regional padel level were recruited by the Andalusian Padel Federation staff team. Players with severe pain or injury in the 6 months before the tests were excluded. All participants followed a similar training routine of 3 days a week disputing 2 regional tournaments per month. After being fully informed of the experimental protocols, all players gave their informed and parental written consent to participate. 

### 2.3. Anthropometric and Body Composition

Stature was measured to the nearest 0.1 cm using a stadiometer (GPM, Seritex Inc., Carlstadt, NJ, USA). Body composition was assessed using a leg-to-leg impedance analyzer (Tanita, BF-350 Ltd., Amsterdam, Netherlands) using the non-athlete mode according to the manufacturer’s guidelines. Measures were collected upon arrival in bare feet and sports clothes (shorts and t-shirt) including a 0.5 kg correction. Participants were instructed to abstain from exercise before their test and avoid caffeinated beverages 24 h before the test. In addition, participants were asked not to drink any fluid 4 h and urinate within 30 min before the tests [34].

### 2.4. Change of Direction and Agility Tests

**Figure 1:**
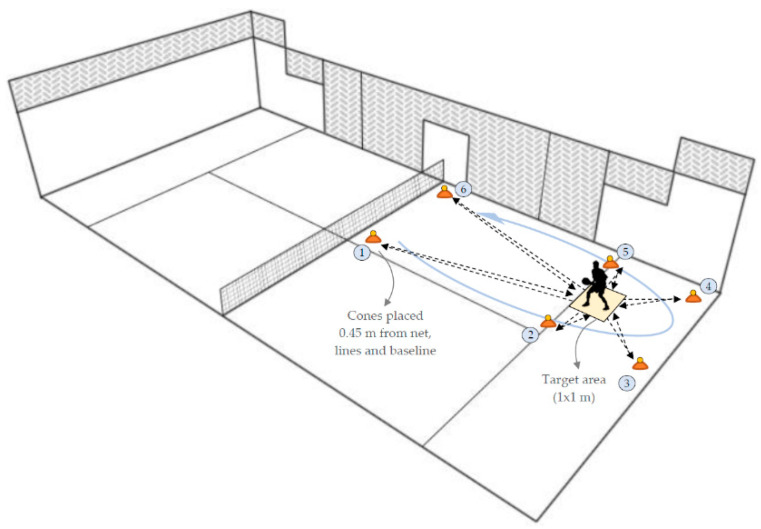
Specific padel agility test. Ref. [27].

Change-of-direction ability and agility were evaluated by two padel-adapted tests [35]. A 3 × 10 m shuttle run test with 180° turn was performed in a turf surface padel court. The fastest of two attempts was considered for the analysis. After 3 min of recovery, players completed a specific padel agility test, originally named the Tapas 6R tests (Figure 1), an agility test specifically designed for padel players assessment [27]. A total of six balls were placed over six flat cones in the depicted specific positions, placed 0.45 m (i.e., the paddle dimensions) from the lines, walls, net, and baseline. Players started in the middle of the service line on a target place of 1 × 1 m in which putting the collected balls. Players were required to run towards each position in the established order, take the ball, turn back to the service position and place it (not dropping) in the target, using the dominant hand to take and place the balls. The time stopped when the last ball is placed. Rate of perceived exertion (RPE) was recorded on the scale of 1 to 10 after each test.

### 2.5. Jumping and Strength Tests

Upper-limb strength measurement included overhead and side medicine ball throws (MBT) with dominant and non-dominant hands, to measure rotational swinging power in forehand and backhand positions [36,37]. Participants were required to throw a 3 kg medicine ball as far and fast as possible. The use of this test has been shown effective to identify isometric maximal trunk rotation torque [38,39]. For the overhead MBT, players stood in a line with their feet side-by-side, facing the throwing direction with the ball back behind the head and threw vigorously forward without moving the feet. For the side MBT, players stood sideways to the starting line and simulated a forehand-backhand stroke tossing the ball as far as possible without crossing the line [28,29]. Each player made two throws per side with 30 s rest. The longest records were collected using a tape measure and selected for further analysis. Following 3 min of recovery, participants completed three attempts separated by 45 s rest for the countermovement jump (CMJ) with their hands on the hips, and the Abalakov vertical jump, following standardized procedures [40]. Maximal vertical jump height was determined using a contact platform (ChronoJump v. 1.9.0, Boscosystem, Barcelona, Spain).

### 2.6. Statistical Analysis

Mean, Standard Deviation, and 95% Confidence Interval (95% CI) of the mean were calculated for each variable. Levene’s test was used to test for equality of variances. Student’s t-test was used to identify gender differences across the fitness variables. One-way ANCOVA was used to determine whether there were significant differences between gender and experience on fitness variables adjusting for age as a covariate as a measure of control for maturity status. Effect sizes (ES) were estimated by partial eta squared, interpreted as small (ES = 0.01), medium (ES = 0.06), and large (ES = 0.14) effects [41]. Sub-analyses were conducted between age and experience groups considering the median (5 years). Level of significance was set at *p* < 0.05 and ES > 0.25. Power analysis was conducted using the G*Power 3.1.9.7 [42]. Statistical calculations were performed using a custom Microsoft Excel spreadsheet and the SPSS v.21 (IBM Corp., Armonk, NY).

## 3. Results

**Figure 2:**
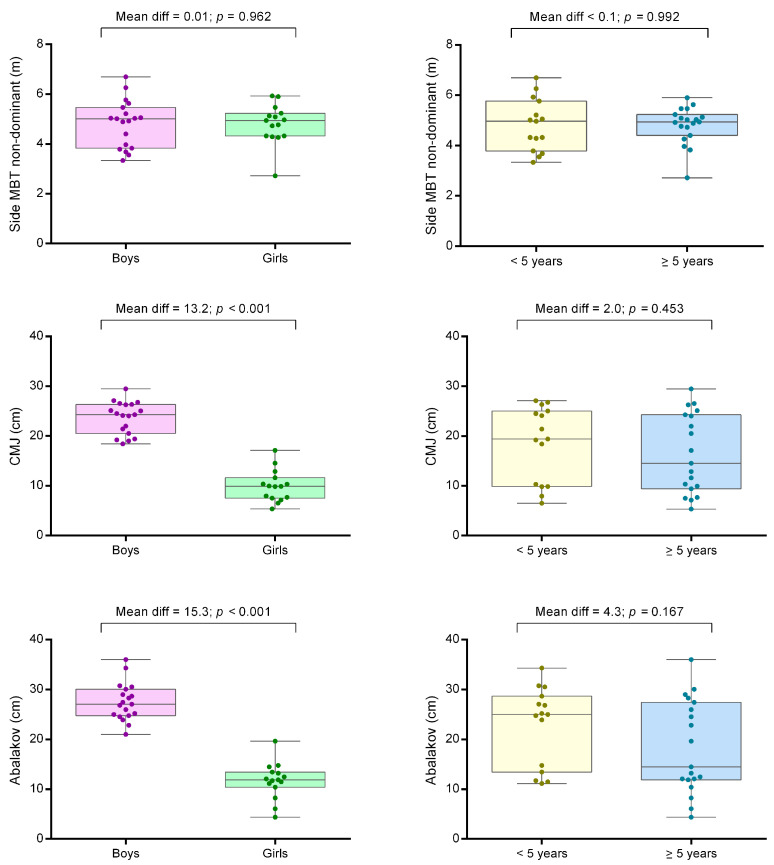
Box and whisker plots of the median (horizontal line), 25th and 75th percentiles (box), range (error bars) and individual scores (markers) for fitness attributes identified as influenced by gender or experience. Left panels are gender group comparisons (boys versus girls). Right panels are the experience groups comparisons considering the median of the sample (lower versus higher than 5 years’ experience). MBT: Medicine ball throw. CMJ: Countermovement jump.

Power analysis determined that the current sample size would allow us to identify significant ANCOVA differences (ES > 0.20; Critical F = 4.171) with a power of 0.81 and an alpha level of 0.05. Physical fitness values of young padel players are presented in Table 1. The influence of gender and experience is shown in Table 2. Figure 2 presents box and whisker plots with posthoc sub-analyses for gender and experience groups. Boys and girls reported a similar practice experience (boys = 6.0 ± 2.7 years, girls = 6.2 ± 2.2 years, *p* = 0.817). There were gender differences on CMJ and Abalakov jumping tests, with boys jumping ~13–15 cm higher (Figure 2). Practice experience affected MBT; however, sub-analysis (i.e., comparisons between players with more or less than 5 years of experience) revealed no significant results (Figure 2). 

## 4. Discussion

This study presents new data about the fitness status of young padel players, boys and girls with different years of practicing experience. In particular, while speed, strength, and agility among young players have been previously described [25], the current study adds information about body composition, jumping ability, and specific tests including side ball medicine ball throws and agility and players change-of-direction ability padel-adapted tests. This novel approach allowed us to identify a similar performance between boys and girls in all fitness tests except for jumps. However, jump disparities may not occur due to the practice of padel but because of natural gender differences at these stages [43]. These results partly contradict earlier gender differences identified among young players using non-specific padel tests [25].

Young padel players described a healthy body composition status (fat mass ~14–18% in boys and ~20–28% in girls and BMI < 25 kg·m^−2^) below BMI and fat mass cut-off points related to health risk [44,45,46]. Certainly, regular padel practice seems not to have an impact on adiposity compared with other racket sports such as tennis [28], badminton [47], or table tennis [48], but might be enough to protect against unhealthy levels of body composition and mitigate the alarming increase in overweight and among youths [10]. Adherence to regular physical activity through childhood is essential to promote healthy growth and development [49]. Of note, evidence supports that physically active youths are likely to maintain a healthy lifestyle in adulthood [50]. Lifelong regular exercise contributes to not just living longer but also in better cardiometabolic and cognitive conditions [51,52] delaying the onset of 40 chronic diseases [53,54,55]. Accordingly, previous studies suggest that regular practice padel is associated with better fitness status in middle-aged adult women [19]. Future longitudinal studies are needed to observe the evolution of fitness status from young to older ages and confirm whether adherence to padel practice in childhood could lead to a healthier adulthood.

Among the options for exercise, our results suggest that padel can constitute an effective strategy for health promotion and increase leisure-time physical activity among sedentary youths [56,57]. Indeed, because padel is characterized by a high rhythm but lower intensity compared to similar racket sports [4,5,6], it might be particularly attractive to encourage adolescents who refuse to practice high-intensity sports to undertake regular physical activity. This hypothesis could be supported by previous studies in adult, recreational padel players who reported enjoyment as the predominant reason for practicing padel, regardless of their age, gender, and experience [8]. Future studies should confirm whereas padel may constitute an attractive physical activity among youths in the leisure time. In addition, considering that Physical Education must be one of the fundamental elements for health promotion in the school population [58], teachers are encouraged to use padel as an easy-to-learn and enjoyable sport to engage youths in physical activity.

Upper- and lower-limbs strength values for young padel players were markedly lower than other racket sports, with poorer throwing and jumping ability [28,47]. These results are in line with earlier studies examining smaller samples of players aged 11 to 16 years [25]. The poorer strength and conditioning profile in padel could be attributable to the lower match activity compared to other racket sports [6]. However, the relationship between fitness status and performance (e.g., are fit players more successful than unfit players?) of injury risks (e.g., are fit players more likely to suffer an injury than unfit players?) are to be explored yet.

This study has some limitations that should be considered. The cross-sectional nature of the data limits the ability to establish cause–effect relationships. Despite not being a broader validated fitness battery, the current field-based tests have been chosen as they are commonly used in padel training, especially in formative stages, making the results highly valuable for practice. In particular, although the specific padel agility test is the only one that has been specifically designed to recreate padel players’ movements, future studies may confirm whereas this test is sensible for effectively measuring agility and corroborate our findings. Results must be interpreted with caution when transferred to other samples of similar age since results might be affected by the maturity stage of participants. Future longitudinal and experimental studies are needed to confirm the potential ability of padel to improve fitness among young players.

The current results have several practical implications such as the use of field-based, padel-adapted tests to measure players’ change-of-direction ability and agility in common distances and movements performed during a game. However, the particularities of this sport would require the use of adapted tests; to the best of our knowledge, there are only two tests specifically designed for padel assessment [27,59]. Therefore, efforts should be made towards studying the design of fitness tests. Likewise, while we did not observe important gender differences in performance, it is essential to maintain this equality in the older stage to guarantee that boys and girls can develop an optimal sports career in equal conditions.

## 5. Conclusions

Male and female young padel players exhibited similar performance in all tests except for jumping ability. Practice experience seemed to influence upper-limb throwing strength; however, sub-analyses revealed no conclusive results. Young padel players presented an apparently healthy body composition status. These results contribute to the existing knowledge in padel by providing new data about the fitness status of amateur young players aged 13 to 17 years old and open a window for future interventions using padel as a health promotion tool among youths.

## Figures and Tables

**Table 1:** Physical fitness and body composition values in boys (*n* = 19) and girls (*n* = 15) padel players aged 13 to 17 years old.

Variable	Boys		Girls		Total	
	M (SD)	95% CI	M (SD)	95% CI	M (SD)	95% CI
Body composition						
Height (cm)	172.8 (8.5)	168.8–176.9	158.6 (3.8)	156.5–160.7	166.6 (9.8)	163.1–170.0
Body mass (kg)	70.2 (14.5)	63.2–77.2	54.7 (9.1)	49.7–59.8	63.4 (14.5)	58.3–68.4
Fat (%)	16.1 (4.8)	13.8–18.4	24.1 (7.5)	20.0–28.2	19.7 (7.2)	17.1–22.2
BMI (kg·m^−2^)	23.3 (3.4)	21.6–25.0	21.7 (3.3)	19.9–23.6	22.6 (3.4)	21.4–23.8
Medicine ball throws (MBT)						
Overhead MBT (m)	6.0 (1.3)	5.4–6.6	5.2 (1.3)	4.5–6.0	5.7 (1.3)	5.2–6.1
Side MBT dominant (m)	5.0 (0.7)	4.7–5.4	4.7 (0.9)	4.2–5.2	4.9 (0.8)	4.6–5.2
Side MBT non-dominant (m)	4.8 (0.9)	4.4–5.3	4.8 (0.8)	4.4–5.2	4.8 (0.9)	4.5–5.1
Jump						
CMJ (cm)	23.2 (4.5)	21.0–25.3	9.9 (3.2)	8.2–11.7	17.3 (7.7)	14.6–20.0
Abalakov (cm)	27.0 (5.0)	24.6–29.4	11.7 (3.6)	9.7–13.7	20.3 (8.9)	17.2–23.4
Change-of-direction/agility						
Padel agility test (s)	18.2 (1.2)	17.7–18.8	19.4 (1.3)	18.7–20.1	18.7 (1.3)	18.3–19.2
RPE padel agility test	5.5 (2.2)	4.5–6.6	4.1 (1.5)	3.3–4.9	4.9 (2.0)	4.2–5.6
Shuttle run 3 × 10 (s)	8.3 (0.4)	8.1–8.5	8.8 (0.6)	8.5–9.1	8.5 (0.6)	8.3–8.7
RPE Shuttle run 3 × 10	5.3 (2.4)	4.1–6.4	4.1 (1.5)	3.2–4.9	4.7 (2.1)	4.0–5.5

BMI: body mass index. CMJ: counter-movement jump. RPE: rate of perceived exertion (1–10 scale). Models adjusted for age as a covariate as a measure of control for maturity status.

**Table 2:** Influence of gender and experience on body composition and physical fitness values in boys (*n* = 19) and girls (*n* = 15) padel players aged 13 to 17 years old.

Fitness Test	Gender		Experience		Gender–Experience	
	*p*-Value	ES	*p*-Value	ES	*p*-Value	ES
Body composition						
Height (cm)	0.253	0.05	0.634	0.01	0.781	<0.01
Weight	0.667	0.01	0.393	0.03	0.792	<0.01
Fat (%)	0.243	0.05	0.326	0.04	0.935	<0.01
BMI (kg·m^−2^)	0.966	<0.01	0.148	0.08	0.895	<0.01
Medicine ball throws (MBT)						
Overhead MBT (m)	0.089	0.09	0.273	0.04	0.160	0.11
Side MBT dominant (m)	0.355	0.03	0.462	0.02	0.540	0.01
Side MBT non-dominant (m)	0.834	<0.01	0.025 *	0.17	0.799	<0.01
Side MBT asymmetry (%)	0.462	0.02	0.585	0.01	0.147	0.08
Jump						
CMJ (cm)	0.031 *	0.16	0.429	0.02	0.316	0.04
Abalakov (cm)	0.019 *	0.19	0.896	<0.01	0.956	<0.01
Change-of-direction/agility						
Padel agility test (s)	0.308	0.04	0.842	<0.01	0.389	0.03
RPE padel agility test	0.308	0.04	0.842	<0.01	0.912	<0.01
Shuttle run 3 × 10 (s)	0.990	<0.01	0.523	0.02	0.649	0.01
RPE Shuttle run 3 × 10	0.801	<0.01	0.090	0.10	0.309	0.04

* Significant between-groups differences (ANCOVA *p* < 0.05). BMI: body mass index. CMJ: counter-movement jump. RPE: rate of perceived exertion (1–10 scale). Gender–Experience: interaction effects showing whether the gender differences are influenced by the practice experience.

## Data Availability

The data presented in this study are available on request from the corresponding author. The data are not publicly available due to privacy.
